# Men and women: beliefs about cancer and about screening

**DOI:** 10.1186/1471-2458-9-431

**Published:** 2009-11-24

**Authors:** Tracey H Sach, David K Whynes

**Affiliations:** 1 School of Chemical Sciences and Pharmacy, University of East Anglia, UK; 2 School of Economics, University of Nottingham, UK

## Abstract

**Background:**

Cancer screening programmes in England are publicly-funded. Professionals' beliefs in the public health benefits of screening can conflict with individuals' entitlements to exercise informed judgement over whether or not to participate. The recognition of the importance of individual autonomy in decision making requires greater understanding of the knowledge, attitudes and beliefs upon which people's screening choices are founded. Until recently, the technology available required that cancer screening be confined to women. This study aimed to discover whether male and female perceptions of cancer and of screening differed.

**Methods:**

Data on the public's cancer beliefs were collected by means of a postal survey (anonymous questionnaire). Two general practices based in Nottingham and in Mansfield, in east-central England, sent questionnaires to registered patients aged 30 to 70 years. 1,808 completed questionnaires were returned for analysis, 56.5 per cent from women.

**Results:**

Women were less likely to underestimate overall cancer incidence, although each sex was more likely to cite a sex-specific cancer as being amongst the most common cancer site. In terms of risk factors, men were most uncertain about the role of stress and sexually-transmitted diseases, whereas women were more likely to rate excessive alcohol and family history as major risk factors. The majority of respondents believed the public health care system should provide cancer screening, but significantly more women than men reported having benefiting from the nationally-provided screening services. Those who were older, in better health or had longer periods of formal education were less worried about cancer than those who had illness experiences, lower incomes, or who were smokers. Actual or potential participation in bowel screening was higher amongst those who believed bowel cancer to be common and amongst men, despite women having more substantial worries about cancer than men.

**Conclusion:**

Our results suggest that men's and women's differential knowledge of cancer correlates with women's closer involvement with screening. Even so, men were neither less positive about screening nor less likely to express a willingness to participate in relevant screening in the future. It is important to understand gender-related differences in knowledge and perceptions of cancer, if health promotion resources are to be allocated efficiently.

## Background

In England, enthusiasm for cancer screening has led to the establishment of organised national programmes, with direct costs being met by the publicly-funded National Health Service (NHS). Since the late-1980s, all women within prescribed age ranges have been invited routinely and regularly for testing for breast cancer and for cervical pre-cancers. The uptake in these two programmes is 75-80 per cent and it is claimed that, over the past 20 years, they have identified around 100,000 breast cancers and around 400,000 significant cervical abnormalities [1]. Whilst routine prostate cancer screening remains to be instituted, eligible men have, since 2002, been entitled to screening tests on demand, subject to accepting information on both the potential benefits and the risks of testing. A national programme for bowel (colorectal) cancer screening, using the faecal occult blood test and including both men and women, was initiated in 2006.

Although health professionals advocate cancer screening on public health grounds, the prospective participant's decision to attend for screening is private. The outcome of that decision depends, in large part, on knowledge, attitudes and concerns with respect both to the disease and to the process of testing. Most of the research into the public's knowledge base conducted to date has been programme-specific and, as a consequence, sex-specific, presumably in view of men's or women's inability to participate directly in one or other of the particular screening services. Typically, only men are asked to provide opinions on screening for prostate cancer and only women are asked for their views on breast screening [2]. Maintaining this restricted perspective seems unwarranted, however. Exclusion from a service on the grounds of incapacity to benefit personally need not entail disinterest. Most individuals of screening age are in female-male partnerships based, presumably, on affection, and one partner's pursuit of an ostensible risk reduction strategy will impact on the emotional wellbeing of the other [3]. Indeed, it is probable that screening decisions in partnerships are actually made jointly rather than individually [4], implying that the attitudes of both sexes are influential, irrespective of whether or not the cancer itself is considered "male" or "female".

This paper reports the findings of a study of men and women's beliefs about, and attitudes towards, cancer and screening. In particular, we were concerned to establish whether men and women differed in their knowledge of risk factors and whether one sex was more favourably disposed towards cancer screening than was the other. As it is traditionally supposed than men are less interested in preventive medicine than are women [5], significant differences in knowledge and attitudes might be anticipated. We also wanted to test, specifically for the case of cancer, the conclusion of a Scottish study of lay beliefs, namely, that each sex perceives itself to be more vulnerable than the opposite sex to major illnesses [6]. We hoped to expand the "scant literature on gender differences in perceived risk and worry for common diseases" ([7] p. 200). In the case of cancer, the literature is based almost exclusively on USA-based surveys, wherein women report higher levels of perceived risk and worry than do men.

## Methods

Our data were obtained via a questionnaire survey [additional file 1], using an instrument designed for self-completion without supervision. This instrument was based on those used in previous local studies of screening knowledge and attitudes [8,9], augmented with questions suggested by other studies of cancer risk perceptions [5,10,11]. After initial construction, the instrument and supporting materials were reviewed for intelligibility by two lay representatives from the Nottingham Primary Care Research Partnership Consumer Panel. The documentation was revised in the light of their observations and suggestions, presented for ethical approval, and revised again accordingly.

As with the previous local studies, we sought the assistance of general practitioners in distributing questionnaires. Two practices, one in Nottingham and one in Mansfield (approximately 12 miles to the north), agreed to send out questionnaires on our behalf. Although both practices were urban, the areas served by each differed in terms of socio-economic deprivation. On the Index of Multiple Deprivation [12], the latter's area was ranked 3,103 amongst the 32,482 enumeration areas of England, whilst the former's was ranked 19,572 (rank 1 = most deprived). The deprivation status of a general practice site generally proves an adequate proxy for deprivation status of the population which it serves [13].

The general practitioners were provided with pre-prepared survey packages, each package containing an invitation letter, a blank questionnaire, a patient information sheet, and a pre-paid response envelope for the return of the completed questionnaire. The practices were asked to mail packages to registered patients aged between 30 and 70 years, but were granted discretion in exclusion. As with the previous studies, patients with a current diagnosis of cancer or with learning disabilities were excluded from the samples, on the grounds of the need to avoid distress and incapacity to complete the instrument, respectively. As the questionnaires were anonymous, we were unable to enhance compliance by contacting non-responders or to identify the non-responders' characteristics. All data were collected in late-2007 and, as far as the results reported here are concerned, all questions were closed-ended (mostly of the tick-box variety).

The opening section of the questionnaire was entitled: "What do you know about cancer?". Subjects were asked, first, to estimate the number of newly-diagnosed cancer cases each year, by selecting one of six numerical values (125,000 to 500,000, in steps of 75,000). As the true number lay between 275,000 and 350,000 [14], values of 125,000 and 200,000 were classified as under-estimates of incidence, and those of 425,000 and 500,000 were classified as over-estimates. Second, subjects were asked to select (in effect, to vote for) "the two most common cancers" in the UK from an alphabetical list of six, namely, bowel (colorectal), breast, cervical, lung, prostate, skin (melanoma). Epidemiologically, the adjective "most common" is ambiguous and might be taken to refer to incidence, to prevalence or to mortality. The imprecision in phrasing was chosen deliberately, however, on the expectation that respondents would be more comfortable with intuitive than with technical phrasing [15]. Whilst the correct answer to this second question is therefore equivocal, the imprecision does not affect our ability to interpret subject response. For some time, the two cancers with the highest incidence have been breast and lung, closely followed by bowel [14]. Lung and bowel are the two most common causes of cancer-related death [16], whilst breast and bowel have the highest prevalences in the population [17]. It follows that, irrespective of interpretation, prostate cancer, melanoma or cervical cancer cannot be considered amongst the "two most common". Indeed, the incidence of cervical cancer is particularly low in England, owing to the effectiveness of the national screening programme.

Subjects were asked to rate each of eight cancer risk factors as a major risk, a minor risk, or as no risk. A "don't know" option was also available. We included increasing age, smoking tobacco and over-indulgence in alcohol use, all of which are generally presumed by health professionals to represent principal risk factors for most forms of cancer. We included being overweight and a lack of exercise, which are known to be risk factors for some of the more common cancers, such as breast and bowel. Sexually-transmitted infection is relevant for a few specific cancers only, although the association between human papillomavirus and cervical cancer is being publicised increasingly. We included two factors which are commonly-cited, although less-well-validated. These were, first, a family history of cancer (genetic risk), even though it "is uncommon for cancer to run in a family... Most of the time, multiple cases of cancer in a family are just a matter of chance" ([18] p.15). Second, we included persistent stress and anxiety, in spite of the association between stress and cancer remaining equivocal [19].

Each subject was offered three true-or-false questions, namely, whether sun-bathing caused skin cancer, whether cancer was more common amongst women than it was amongst men, and whether more people died of heart disease than died of cancer. The first statement, of course, has long been accepted as true by both practitioners and public alike [20,21] and we expected most, if not all, subjects to respond accordingly. The numbers of males and of females dying from cancer each year are approximately equal, as are the numbers of new cases diagnosed. Age-standardised rates for males, however, have long been significantly higher than those for females [22]. The relative importance of cancer and heart disease is a question of interpretation: "At all ages, there are more deaths from cancer than from ischaemic heart disease. However, no single cancer is a more common cause of death than ischaemic heart disease" ([23] p.16). It should be stressed that the study was less concerned with subjects being objectively wrong or right in their knowledge and more concerned with the existence of differences in response between the sexes.

Three types of cancer screening currently available via the NHS - bowel, breast and prostate - were described briefly. We restricted discussion to these three types, on the grounds that men and women would each be eligible for two types only. We asked the respondents whether they believed that the NHS should continue to provide screening services such as these. They were also asked to indicate whether they had already been screened, or whether or not they would accept a test were one to be offered.

The final section of the questionnaire was modelled closely on the instruments used in previous local studies [8,9]. It requested socio-demographic information including sex, age, marital status, and age on leaving full-time education. Ethnic origin was to be identified as one of African, Afro-Caribbean, Asian, Chinese, White or "other". Annual household income was represented by a choice of one from four income bands, beginning at 0-£10,000 and thereafter in increments of £10,000 to £30,000 and above. An individual with average household income in and around the Nottingham would have selected the £20-30,000 band [24]. We requested an indication of smoking status (current smoker, ex-smoker, never smoked) and subjects' perception of their weight (over-, under-, about right). Subjects reported illness experiences, i.e. whether they or a close family member had ever suffered from each of six conditions (stomach complaints, piles or haemorrhoids, heart disease, cancer, stroke, depression). We invited subjects to indicate their degree of worry about the prospect of cancer (4-point scale: not worried, minor concerns, quite worried, very worried). To assess personal risk perception, we invited respondents to assess their own chances of getting cancer, relative to people of their own age and sex (5-point scale: much less, less, the same, more, much more). Subjects described their current states of health by completing standard EQ-5D (EuroQoL) health state classifications [25]. Classified states were translated into EQ index scores using to the current UK tariff [26].

We planned two types of analysis. First, responses to the factual questions, such as estimated incidence, identity of most common cancers and risk factors, were examined with respect to male-female differences in proportions. Beyond establishing whether men's and women's judgements were accurate, we were concerned to establish whether opinions differed systematically by sex. Second, three specific beliefs or attitudes about cancer and screening were modelled using logistic regressions, again with a view of establishing the existence, or otherwise, of male-female differences. The dependent variables in the regressions were (i) the belief that the chances of getting cancer were above average, (ii) having substantial concerns about cancer, and (iii) actual or potential participation in bowel cancer screening. We restricted the analysis of participation to bowel screening, as this was the only type for which both sexes were eligible. All of the socio-demographic and knowledge variables were candidates for the models initially, which were re-estimated after excluding variables with insignificant coefficients.

## Results

Out of 6,939 questionnaires distributed, 1,808 were completed and returned for analysis. The response rate (26.1 per cent) was similar to that achieved in a previous local study [9]. Of those questionnaires returned, 1,016 (56.5 per cent) had been completed by women. Table 1 displays the socio-demographic characteristics of the sample. The male and female sub-samples did not differ significantly with respect to years of full-time education, ethnicity, cohabitation status, current health (EQ index score) and weight perception. Women, however, were younger on average, and were more likely to drawn from lower income households. Significantly more women than men had illness experiences, for all six of the conditions nominated. Although the proportion of current smokers was similar between sub-samples, a higher proportion of women had never smoked.

**Table 1:** Socio-demographic characteristics

		Women	Men	t or χ^2^	p =
Mean age, years		49.7	52.3	-4.88	0.00
Mean age leaving full-time education, years		17.4	17.4	-0.18	0.86
Ethnicity, % white		97.7	96.9	1.17	0.28
Married or with a partner, %		77.7	80.3	1.86	0.17
Annual household income, %	Up to £10,000	18.4	16.3	10.24	0.02
	£10-20,000	29.4	23.9		
	£20-30,000	16.8	19.1		
	Above £30,000	35.3	40.6		
Mean EQ index score		0.82	0.83	-0.81	0.42
Illness experience, %	Stomach problems	41.2	35.1	7.02	0.01
	Piles/haemorrhoids	48.8	61.8	30.19	0.00
	Heart disease	44.8	37.8	8.89	0.00
	Cancer	62.1	46.7	42.00	0.00
	Stroke	34.1	29.6	4.12	0.04
	Depression	49.1	34.8	36.53	0.00
Tobacco smoking, %	Current smoker	14.8	14.0	36.67	0.00
	Ex-smoker	27.8	41.2		
	Never smoked	56.5	43.5		
Considers self to be overweight, %		39.7	38.8	0.15	0.70

Table 2 displays the responses to the first two factual questions. Identifying the "most common" cancers by means of a simple count of subjects' votes (two per subject) produced an overall ranking broadly consistent with any of the interpretations of the phrase, namely, breast, bowel, lung, prostate, skin and cervical. Even so, more than one-quarter of the votes (27.9 per cent) were cast for cancers which could not be considered common by any interpretation. Comparing the male/female sub-samples, equal proportions of men and women believed breast, lung or skin cancer to be amongst the two most common. However, a significantly higher proportion of women believed bowel or cervical cancer to be one of the two most common, whereas a significantly higher proportion of men believed prostate cancer to be one of the two most common. Proportionately fewer women than men under-estimated overall cancer incidence.

**Table 2:** Beliefs about prevalence and incidence

	**% of votes cast**	**% voting for each cancer as one of the two most common:**			
		**Women**	**Men**	**χ^2^**	**p =**
	
Two most common cancers					
Bowel	19.0	41.3	32.9	13.24	0.00
Breast	37.5	74.7	73.6	0.32	0.57
Cervical	4.2	10.1	6.0	9.41	0.00
Lung	15.6	30.3	31.8	0.48	0.49
Prostate	14.1	21.6	35.9	44.97	0.00
Skin (melanoma)	9.6	19.3	18.7	0.10	0.75
Estimated cancer incidence					
Over-estimate		34.7	33.2	5.93	0.05
Correct		44.2	40.7		
Under-estimate		21.2	26.1		

Table 3 presents the proportion of subjects' declaring an inability to judge the level of risk represented by the various risk factors. The factors about which most subjects were uncertain (i.e. the highest proportion of "don't know" responses in assigning a risk level) were stress and sexually-transmitted diseases, significantly more so for men in the case of the latter. Scientifically speaking, the response was appropriate in the first case but less so in the second. The risk factors about which most subjects expressed certainty (i.e. the lowest proportion of "don't know" responses) were tobacco smoking and family history, significantly less so for men in the case of the latter. Only one individual responded "don't know" to all eight risk factors.

**Table 3:** Proportion (%) unable to assign a level of risk to a risk factor

**Risk factor**		**%**	**χ^2^**	**p =**
	
Being overweight	Women	10.7	0.10	0.76
	Men	11.2		
Smoking tobacco	Women	0.0	2.59	0.11
	Men	0.3		
Too much alcohol	Women	7.1	0.05	0.82
	Men	6.8		
Lack of exercise	Women	11.0	0.74	0.39
	Men	9.8		
Sexually-transmitted diseases	Women	13.9	6.32	0.01
	Men	18.3		
Family history/genetic	Women	1.0	9.21	0.00
	Men	2.9		
Increasing age	Women	7.3	1.13	0.29
	Men	6.0		
Stress and anxiety	Women	16.3	0.80	0.37
	Men	14.8		

Table 4 indicates risk assessments for those who felt able to judge. For the whole sample, the ranking of potential factors dismissed as irrelevant to cancer risk were, in descending order, lack of exercise, stress, sexually-transmitted disease, being overweight, age, alcohol, family history, smoking. The risk factors considered to be major by the most subjects were smoking, excessive alcohol consumption and family history, significantly more so by women in the case of the last two. Compared with men, women assigned a higher risk status to sexually-transmitted diseases, but there were no differences of opinion between men and women as regards weight, exercise, age and stress. The most common (n = 35) combination of responses to the risk factor questions was to identify all as major risks, closely followed (n = 28) by assigning smoking, family history and age as major risks, with the remainder as minor risks.

**Table 4:** Level of risk assigned (% of those assigning a level)

**Risk factor**		**Major**	**Minor**	**None**	**χ^2^**	**p =**
		
Being overweight	Women	32.3	53.8	13.8	0.77	0.68
	Men	31.6	53.0	15.4		
Smoking tobacco	Women	97.4	2.6	0.0	0.13	0.72
	Men	97.7	2.3	0.0		
Too much alcohol	Women	48.9	42.2	8.9	11.55	0.00
	Men	40.6	48.0	11.3		
Lack of exercise	Women	15.8	58.5	25.7	2.75	0.25
	Men	15.4	55.2	29.4		
Sexually-transmitted diseases	Women	36.7	47.5	15.8	44.48	0.00
	Men	23.2	49.7	27.1		
Family history/genetic	Women	77.7	22.0	0.3	21.27	0.00
	Men	71.2	26.4	2.4		
Increasing age	Women	33.8	52.7	13.5	3.86	0.14
	Men	36.7	52.8	10.5		
Stress and anxiety	Women	25.1	53.7	21.2	1.46	0.48
	Men	25.7	50.8	23.4		

With respect to the three true-or-false questions, nearly all subjects (98.7 per cent) agreed that sun-bathing was a skin cancer risk. The majority (53.3 per cent) did not accept that cancer was more common amongst women than it was amongst men, although 26.2 per cent reported "don't know". Two-fifths of the sample (41.5 per cent) thought (incorrectly) that more people died of heart disease than died of cancer, with 25.9 per cent reporting "don't know". For none of these questions did the proportions of male and female responses differ significantly (χ^2 ^test, p = 0.21 or greater). A "don't know" response to both of the last two questions was offered by 10.1 per cent of the sample, with no significant difference between the sexes (χ^2 ^= 0.01, p = 0.92).

Virtually the entire sample (99.6 per cent) believed that cancer screening services should continue to be provided by the National Health Service. For breast cancer screening, 47.4 per cent of women reported that they had already been screened, and a further 48.4 per cent indicated that they would take the test, were one to be offered. The corresponding proportions for prostate cancer screening amongst men were 7.4 per cent and 83.6 per cent, respectively. The higher rate of actual participation in breast cancer screening is to be expected, in view of the longer duration and higher public profile of that programme. Combining the actual and potential participation percentages, the proportion of women positively receptive to breast cancer screening was higher than the proportion of men receptive to prostate cancer screening (95.8 vs. 91.0 per cent, Z = 4.11, p < 0.01). Interest in bowel cancer screening was slightly lower, with 6.8 per cent of men and women reporting a previous screen, and a further 80.3 per cent indicating a wish to be tested. Most people in the sample (69.6 per cent) assessed their chances of getting cancer as the same as the average for their age and sex, although 15.3 and 15.1 per cent believed that their chances were higher and lower, respectively. Most subjects (75.5 per cent) recorded either no or minor worries about cancer, the remainder having more substantial concerns (quite or very worried).

Tables 5, 6 and 7 present the final versions of the three logistic regression models. Being older or in better health made individuals less likely to have substantial worries about cancer or to perceive their cancer risk to be above average. Those with longer periods of formal education were also less likely to have cancer worries. Illness experiences, especially an experience of cancer, increased the likelihoods of a perceived higher risk, cancer worries and enthusiasm for bowel cancer screening. Correspondingly, those expressing no worries about cancer were less-favourably disposed towards bowel screening. Those from lower income bands were more likely to be worried about cancer but less likely to embrace screening. Smokers saw themselves to be at higher risk of cancer, especially if they believed lung cancer to be common, although they were comparatively unenthusiastic about bowel screening. As might have been anticipated, under-estimating cancer incidence reduced the likelihood of seeing oneself to be at risk, whilst believing bowel cancer to be common increased the likelihood of being favourably disposed towards bowel screening. Those considering themselves overweight believed they faced a greater cancer risk. Finally, women were more likely than men to have substantial worries about cancer, yet were less likely to be favourably disposed towards bowel screening. It should be noted that, for none of the models, were the coefficients associated with ethnicity, marital status and general practice of origin statistically significant.

**Table 5:** Logistic regression: Chances of getting cancer believed to be above average = 1

**Variables**	**β**	**Odds ratio**	**95% CI**
	
Constant	-1.14		
Age, years	-0.03	0.97	0.96 - 0.98
Experience: stomach problems = 1	0.29	1.34	1.00 - 1.80
Experience: heart disease = 1	0.31	1.36	1.01 - 1.83
Experience: cancer = 1	1.49	4.46	3.12 - 6.37
EQ index score	-0.90	0.41	0.23 - 0.73
Current smoker = 1	1.17	3.21	2.24 - 4.59
Overweight = 1	0.58	1.78	1.32 - 2.40
Cancer incidence under-estimated = 1	-0.45	0.64	0.44 - 0.93
Smoking and lung cancer* = 1	0.60	1.83	1.25 - 2.67
Nagelkerke R^2^	0.22		

* those with a history of smoking who believe lung cancer to be amongst the two most common.

**Table 6:** Logistic regression: Quite or very worried about cancer = 1

**Variables**	**β**	**Odds ratio**	**95% CI**
	
Constant	2.43		
Sex, male = 1	-5.15	0.60	0.46 - 0.77
Age, years	-0.04	0.97	0.96 - 0.98
Age leaving full-time education, years	-0.07	0.93	0.89 - 0.98
Income, £10-20,000 = 1	0.37	1.45	1.11 - 1.90
Experience: haemorrhoids = 1	0.26	1.30	1.02 - 1.66
Experience: heart disease = 1	0.26	1.29	1.01 - 1.65
Experience: cancer = 1	0.48	1.62	1.26 - 2.08
EQ index score	-1.25	0.29	0.17 - 0.48
Nagelkerke R^2^	0.12		

**Table 7:** Logistic regression: Has accepted, or would accept, an offer to be screened for bowel cancer = 1

**Variables**	**β**	**Odds ratio**	**95% CI**
	
Constant	1.65		
Sex, male = 1	0.57	1.76	1.28 - 2.42
Income, < £10,000 = 1	-0.97	0.38	0.27 - 0.53
Experience: stomach problems = 1	0.50	1.65	1.19 - 2.28
Experience: cancer = 1	0.46	1.59	1.17 - 2.16
Current smoker = 1	-0.53	0.59	0.40 - 0.86
Bowel cancer believed to amongst the two most common = 1	0.49	1.64	1.18 - 2.27
No worries about cancer = 1	-0.76	0.47	0.33 - 0.65
Nagelkerke R^2^	0.10		

## Discussion

Our results did not confirm fully the hypothesis that each sex feels itself to be more vulnerable than the opposite sex to cancer [6]. In our sample, men and women responded in approximately equal proportions when asked directly whether cancer was more common amongst members of one sex than amongst those of the other. Neither sex believed disproportionately that cancer was more or less destructive than heart disease. This having been said, women were more likely to suggest cervical cancer (female-specific) as one of the common types, whereas men were more likely to suggest prostate cancer (male-specific). As strict factual accuracy would have required neither to have been selected, the inaccuracies themselves are consistent with the vulnerability hypothesis. The belief amongst women that cervical cancer is widespread in England has been observed before [27] and can be ascribed in part to the social amplification of risk engendered by the national screening programme [9]. Bowel cancer featured less prominently amongst men's rankings than it did amongst women's in our sample. Bowel cancer is much less common than prostate cancer within the set of cancers to which men specifically are susceptible, although it and lung cancer would rank in second place for specifically-female cancers. This having been said, bowel cancer in England has a higher incidence amongst men than it has amongst women.

Although there exist few other both-sex studies of beliefs about cancer in general, several single-sex studies or both-sex studies of one or more cancer types have been undertaken. Several of our risk factor findings parallel those of earlier studies. First, age was under-rated as a risk factor for cancer by both men and women equally, being assessed as a major risk only by around one-third of subjects. An earlier British population study [10] also reported a comparative disregard for age as a risk, as did studies of specific cancer types, such as breast [28] and bowel [29]. Second, particular prominence was accorded to the family history and the genetic basis of cancer, particularly by women. A similar finding was reported by an American population study [30] and a British study of bowel cancer awareness [31]. Family history was the most frequently chosen risk in women-only studies of cervical cancer [9,32,33], although it is evident that such a belief can prevail amongst men also [34]. Third, high perceived risks from smoking were also reported equally for both sexes in the cited British population study [10], whilst those posed by both alcohol and smoking were similarly reported in an English study organised, like ours, via general practice [27].

Where the assignment of a risk factor's severity differed significantly by sex (Table 4), women rated that factor more highly, and similar finding was reported in a Japanese population study [35]. In the Japanese sample, infections, smoking, stress and endocrine-disrupting chemicals (EDCs) were rated as the main sources of risk, with lack of exercise, being overweight and alcohol being considered relatively unimportant. As this particular study was concerned with knowledge of avoidable risk, age and family history were not available to be identified as risk factors. The authors ascribed the precedence given to infections and chemicals to intense recent media coverage of both EDCs and a contemporary epidemic of Sudden Acute Respiratory Syndrome. By the same token, it is possible that the recognition of alcohol and sexually-transmitted disease risk by women in our sample follows from the English media's sustained coverage of the health consequences of the supposedly-upward trend in drinking and sexual promiscuity amongst younger women [36].

The earlier British study [10] included so-called "mythic causes" of cancer (food additives, proximity to overhead power lines, pollution and stress) in its range of risk factor options, "mythic causes" being behaviours or sets of circumstances whose associations with cancer remain to be established scientifically. Leaving aside the question as to whether or not such factors genuinely are mythic, relatively few subjects in that study were convinced of the importance of the first three, although stress was cited by up to 25 per cent of respondents. In our own study, over 60 per cent of both men and women considered stress to be a risk factor. Stress was considered an important risk factor in the Japanese sample [35], as it was by subjects in an Irish study of breast cancer [37]. In none of these studies was a differential response by gender reported. However, when actual cancer patients in the USA were asked to assign causes for their condition, stress was posited as a causal factor by around 20 per cent of men but by around 40 per cent of women [38].

Our regression analysis suggested that comparative risk perception was positively associated with illness experiences, smoking and being overweight, negatively associated with age and the current level of general health, and unrelated to gender. This result merits comparison with those of two USA population studies. First, a regression analysis of routinely-collected national socio-demographic and health data indicated that a high perceived **absolute **risk of cancer was negatively associated with age and positively associated with current smoking, alcohol consumption, having one or more relatives with cancer, low income, with being obese (but not with being overweight), and with being female [39]. In the second study [40], high perceived **relative **risk was negatively associated with age and with general health, and positively associated with a family history of cancer and being male. In so far as the American findings can be pooled, they seem to mirror our finding that current health status, family cancer, smoking and age are robust predictors of risk perception, whilst the significance of gender remains equivocal.

The variables in our model predicting heightened worries about cancer (having a family experience of cancer, being less educated, being younger or in poorer health, and being female) are also reported as significant in the analysis and attendant literature review of the second US study cited above [40]. Neither analysis included family experiences of illness other than cancer which emerged as significant predictors of worry in our model. Our findings are consistent with those of a US study [7] concluding that women worry more about cancer than do men, and with those of a study reporting that worry about, and perceived risk of, one serious disease such as cancer, heart disease or diabetes is influenced positively by the presence of both that disease and other such serious diseases amongst family members [11].

Contrary to the supposition that men are less interested in preventive medicine than are women [5], almost everyone in our study, irrespective of sex, agreed that NHS should be providing cancer screening services. Such widespread agreement might be a reflection of the way health care is financed in England; all individuals contribute to the NHS via tax contributions related to ability to pay and unrelated to whether or not health care is actually consumed. Thus, men have always contributed to the costs women's care services, and vice versa. Despite men's interest in prostate cancer screening being lower than women's interest in breast cancer screening, more than 90 per cent remained favourably disposed towards testing. The disparity in interest by sex must be explicable in part by the weight of publicity attached to the national breast screening programme in England, contrasting with the NHS's deliberately-discreet approach towards prostate screening. Indeed, it is probable that men are currently over-receptive to prostate cancer screening. Men-only studies both in Sweden [41] and in England and Wales [42] show that knowledge levels about the disease and the consequences of screening are typically low, although attitudes towards testing are uniformly positive. Health education, in the form of the provision of authoritative information, significantly reduces the propensity to demand prostate screening. Ostensibly countering the stereotype, our logistic regression result predicts that an interest in bowel cancer screening is more probable amongst men. Although there is some US evidence to support of this finding [43], it is inconsistent with that of another English survey [44] and also with English experience prior to the recent implementation of the national faecal occult blood screening programme. Participation was significantly higher amongst women than amongst men in both the English randomised controlled trial [45] and the programme's pilot phase [46].

Our study has limitations. We elected to distribute questionnaires via general practitioners rather than by direct mailing. Experience has shown that this method achieves a much higher response rate, albeit at the risk of introducing bias. In the absence of detailed information on each subject offered a questionnaire, response bias by characteristic cannot be ascertained. Despite guidance from the investigators, practitioners' exclusions of potential respondents remained discretionary and unrecorded, although it is probable that the sample excluded a disproportionate number of subjects with cognition difficulties or with particularly close contacts with cancer. This having been said, detailed analyses of respondents in local bowel [47] and cervical [9] screening studies, which also used the general practice distribution method, failed to provide evidence of systematic differences in characteristics between samples and populations. Of possibly more substance with respect to possible bias concerns the findings with respect to bowel cancer. The Nottingham area has hosted the national clinical trial of bowel screening since the 1980s. The trial has never been widely publicised, yet it's very scale must have familiarised many local people with the condition and the procedure. It is therefore probable that Nottingham's views on bowel cancer are not, strictly speaking, representative of those of rest of the country.

## Conclusion

The development of national cancer screening services in England has strongly favoured women's health promotion opportunities compared with those of men's, owing to both biology and technology. Not surprisingly, in view of the nature of service provision, far fewer men than women in our sample had experienced screening. Given women's greater involvement in the process, therefore, we had anticipated that knowledge of cancer and attitudes towards screening would vary with sex. Compared with women, men were more likely to underestimate cancer incidence. Whilst neither sex believed themselves to be more susceptible to cancer **per se**, each did believe a same-sex-specific cancer to be amongst the most common types. With respect to sexually-transmitted infections and family history as risk factors, men were more likely to admit ignorance, and less likely to perceive each as a major risk. In reality, each of these factors is more significant for female-specific cancers (notably, cervical and breast, respectively). Overall, our results suggest that knowledge of cancer correlates with women's closer involvement with screening. This having been said, men were neither less positive about screening nor less likely to express a willingness to participate in relevant screening in future, suggesting that personal experience alone fails to explain attitudes. Although women expressed more worries about cancer, men expressed the greater interest in bowel cancer screening.

Given differential knowledge, it is inevitable that differences exist between the sexes in the process of reaching screening decisions. In the past, health education and health promotion programmes have remained "gender blind", yet presuming gender or sex to be irrelevant to how people perceive and comprehend is a potential obstacle to the development of effective health promotion policies [48]. International organisations are emphasising gender as both fundamental and integral to health care analysis and policy [49], and the findings of this study endorse the importance of research into gender-based inequalities in the provision of cancer services in England [50].

## Competing interests

The authors declare that they have no competing interests.

## Authors' contributions

The authors contributed equally to the design of the study, data analysis and the drafting of the manuscript. THS organised the data collection and was responsible for data entry.

## Pre-publication history

The pre-publication history for this paper can be accessed here:

http://www.biomedcentral.com/1471-2458/9/431/prepub

## Supplementary Material

Additional file 1 **Questionnaire: Men and women: beliefs about cancer and about screening**. Content of the questionnaire used for data capture. Click here for file

## References

[B1] Patnick J Ed Breast and cervical screening: the first 20 years 2008 Sheffield: NHS Cancer Screening Programmes

[B2] Gigerenzer G Mata J Frank R Public knowledge of benefits of breast and prostate cancer screening in Europe Journal of the National Cancer Institute 2009 101 17 1216 1220 10.1093/jnci/djp237 19671770 PMC2736294

[B3] Baider L Bengel J Cancer and the spouse: gender-related differences in dealing with health care and illness Critical Reviews in Oncology/Hematology 2001 40 115 123 10.1016/S1040-8428(01)00137-8 11682318

[B4] Umberson D Gender, marital status and the social control of health behaviour Social Science and Medicine 1992 34 8 907 917 10.1016/0277-9536(92)90259-S 1604380

[B5] Evans REC Brotherstone H Miles A Wardle J Gender differences in early detection of cancer Journal of Men's Health and Gender 2005 2 2 209 217 10.1016/j.jmhg.2004.12.012

[B6] Macintyre S McKay L Ellaway A Who is more likely to experience common disorders: men, women, or both equally? Lay perceptions in the West of Scotland International Journal of Epidemiology 2005 34 461 466 10.1093/ije/dyh333 15737979

[B7] Wang C O'Neill SM Rothrock N Gramling R Sen A Acheson LS Rubinstein WS Nease DE Jr Ruffin MT IV the Family Healthware™ Impact Trial (FHITr) group Comparison of risk perceptions and beliefs across common chronic diseases Preventive Medicine 2009 48 197 202 10.1016/j.ypmed.2008.11.008 19073208 PMC2720609

[B8] Whynes DK Frew E Wolstenholme JL A comparison of two methods for eliciting contingent valuations of colorectal cancer screening Journal of Health Economics 2003 22 4 555 574 10.1016/S0167-6296(03)00006-7 12842315

[B9] Philips Z Avis M Whynes DK Knowledge of cervical cancer and screening among women in east-central England International Journal of Gynecological Cancer 2005 15 639 645 10.1111/j.1525-1438.2005.00126.x 16014118

[B10] Wardle J Waller J Brunswick N Jarvis MJ Awareness of risk factors for cancer among British adults Public Health 2001 115 173 174 10.1016/S0033-3506(01)00439-5 11429711

[B11] DiLorenzo TA Schnur J Montgomery GH Erblich J Winkel G Bovbjerg DH A model of disease-specific worry in heritable disease: the influence of family history, perceived risk and worry about other illnesses Journal of Behavioral Medicine 2006 29 1 37 49 10.1007/s10865-005-9039-y 16470344

[B12] Noble M McLennan D Wilkinson K Whitworth A Barnes H Dibben C The English indices of deprivation 2007 2008 London: Department for Communities and Local Government

[B13] Strong M Maheswaran R Pearson T A comparison of methods for calculating general practice level socioeconomic deprivation International Journal of Health Geographics 2006 5 29 16820054 10.1186/1476-072X-5-29 PMC1524946

[B14] Statistical Information Team CancerStats Report: Incidence, UK 2008 2008 London: Cancer Research UK

[B15] Chapman K Abraham C Jenkins V Fallowfield L Lay understanding of terms used in cancer consultations Psycho-Oncology 2003 12 557 566 10.1002/pon.673 12923796

[B16] Statistical Information Team CancerStats Report: Mortality, UK 2007 London: Cancer Research UK

[B17] Forman D Stockton D Møller H Quinn M Babb P De Angelis R Micheli A Cancer prevalence in the UK: results from the EUROPREVAL study Annals of Oncology 2003 14 648 654 10.1093/annonc/mdg169 12649115

[B18] National Cancer Institute What you need to know about cancer 2005 Rockville, MD: National Cancer Institute

[B19] Metcalfe C Davey Smith G Macleod J Hart C The role of self-reported stress in the development of breast cancer and prostate cancer: a prospective cohort study of employed males and females with 30 years of follow-up European Journal of Cancer 2007 43 1060 1065 10.1016/j.ejca.2007.01.027 17336053

[B20] Albert MR Ostheimer KG The evolution of current medical and popular attitudes toward ultraviolet light exposure: part 3 Journal of the American Academy of Dermatology 2003 49 1096 1106 10.1016/S0190-9622(03)00021-5 14639391

[B21] Peacey V Steptoe A Sanderman R Wardle J Ten-year changes in sun protection behaviors and beliefs of young adults in 13 European countries Preventive Medicine 2006 43 460 465 10.1016/j.ypmed.2006.07.010 16949656

[B22] Westlake S Cooper N Cancer incidence and mortality: trends in the United Kingdom and constituent countries, 1993 to 2004 Health Statistics Quarterly 2008 38 33 46 18595386

[B23] Griffiths C Rooney C Brock A Leading causes of death in England and Wales - how should we group causes? Health Statistics Quarterly 2005 28 6 17 16315552

[B24] MacSearraigh E Marais J Schuster S Regional household income Economic Trends 2006 633 29 63

[B25] Brooks R Rabin R de Charro F Eds The measurement and valuation of health status using EQ-5D: a European perspective 2003 Dordrecht: Kluwer Academic Publishers

[B26] Dolan P Modeling valuations for EuroQoL health states Medical Care 1997 35 11 1095 1108 10.1097/00005650-199711000-00002 9366889

[B27] Adlard JW Hume MJ Cancer knowledge of the general public in the United Kingdom: survey in a primary care setting and review of the literature Clinical Oncology 2003 15 174 180 10.1016/S0936-6555(02)00416-8 12846494

[B28] Chamot E Perneger TV Men's and women's knowledge and perceptions of breast cancer and mammography screening Preventive Medicine 2002 34 380 385 10.1006/pmed.2001.0999 11902856

[B29] McCaffery K Wardle J Waller J Knowledge, attitudes, and behavioral intentions in relation to the early detection of colorectal cancer in the United Kingdom Preventive Medicine 2003 36 525 535 10.1016/S0091-7435(03)00016-1 12689797

[B30] Breslow RA Sorkin JD Frey CM Kessler LG Americans' knowledge of cancer risk and survival Preventive Medicine 1997 26 171 177 10.1006/pmed.1996.0136 9085385

[B31] Robb KA Miles A Wardle J Demographic and psychosocial factors associated with perceived risk for colorectal cancer Cancer Epidemiology, Biomarkers and Prevention 2004 13 3 366 372 15006910

[B32] Baay MFD Verhoeven V Avonts D Vermorken JB Risk factors for cervical cancer development: what do women think? Sexual Health 2004 1 145 149 10.1071/SH04004 16335302

[B33] Waller J McCaffery K Wardle J Beliefs about the risk factors for cervical cancer in a British population sample Preventive Medicine 2004 38 745 753 10.1016/j.ypmed.2004.01.003 15193894

[B34] Verhoeven V Baay M Colliers A Verster A Van Royen P Avonts D Vermorken JB The male factor in cervical carcinogenesis: a questionnaire study of men's awareness in primary care Preventive Medicine 2006 43 389 393 10.1016/j.ypmed.2006.06.006 16872669

[B35] Inoue M Iwasaki M Otani T Sasazuki S Tsugane S Public awareness of risk factors for cancer among the Japanese general population: a population-based survey BMC Public Health 2006 6 2 10.1186/1471-2458-6-2 16403223 PMC1351169

[B36] Jackson C Tinkler P 'Ladettes' and 'Modern Girls': 'troublesome' young femininities Sociological Review 2007 55 2 251 272 10.1111/j.1467-954X.2007.00704.x

[B37] McMenamin M Barry H Lennon A-M Purcell H Baum M Keegan D McDermott E O'Donoghue D Daly L Mulcahy H A survey of breast cancer awareness and knowledge in a Western population: lots of light but little illumination European Journal of Cancer 2005 41 393 397 10.1016/j.ejca.2004.11.015 15691638

[B38] Wold KS Byers T Crane LA Ahnen D What do cancer survivors believe causes cancer? Cancer Causes and Control 2005 16 115 123 10.1007/s10552-004-2414-0 15868453

[B39] Honda K Neugut AI Associations between perceived cancer risk and established risk factors in a national community sample Cancer Detection and Prevention 2004 28 1 7 10.1016/j.cdp.2003.12.001 15041071

[B40] McQueen A Vernon SM Meissner HI Rakowski W Risk perceptions and worry about cancer: does gender make a difference? Journal of Health Communication 2008 13 56 79 10.1080/10810730701807076 18307136

[B41] Berglund G Nilsson S Nordin K Intention to test for prostate cancer European Journal of Cancer 2005 41 990 997 10.1016/j.ejca.2005.01.011 15862747

[B42] Watson E Hewitson P Brett J Bukach C Evans R Edwards A Elwyn G Cargill A Austoker J Informed decision making and prostate specific antigen (PSA) testing for prostate cancer: a randomised controlled trial exploring the impact of a brief patient decision aid on men's knowledge, attitudes and intention to be tested Patient Education and Counseling 2006 63 367 379 10.1016/j.pec.2006.05.005 16875796

[B43] Friedemann-Sanchez G Griffin JM Partin MR Gender differences in colorectal cancer screening barriers and information needs Health Expectations 2007 10 148 160 10.1111/j.1369-7625.2006.00430.x 17524008 PMC5060384

[B44] Taskila T Wilson S Damery S Roalfe A Redman V Ismail T Hobbs R Factors affecting attitudes toward colorectal cancer screening in the primary care population British Journal of Cancer 2009 101 250 255 10.1038/sj.bjc.6605130 19550423 PMC2720207

[B45] Hardcastle JD Chamberlain JO Robinson MHE Moss SM Amar SS Balfour TW James PD Mangham CM Randomised controlled trial of faecal-occult-blood screening for colorectal cancer The Lancet 1996 348 1472 1477 10.1016/S0140-6736(96)03386-7 8942775

[B46] Weller D Coleman D Robertson R Butler P Melia J Campbell C Parker R Patnick JS Moss S The UK colorectal cancer screening pilot: results of the second round of screening in England British Journal of Cancer 2007 97 1601 1605 10.1038/sj.bjc.6604089 18026197 PMC2360273

[B47] Frew EJ Hammersley V Wolstenholme JL Whynes DK Collaborating with a primary care-based research network Journal of Evaluation in Clinical Practice 2001 7 3 339 342 10.1046/j.1365-2753.2001.00292.x 11555092

[B48] Ostlin P Eckermann E Mishra US Nkowane M Wallstam E Gender and health promotion: A multisectoral policy approach Health Promotion International 2007 21 S1 25 35 10.1093/heapro/dal048 17307954

[B49] Office of the Special Adviser on Gender Issues and Advancement of Women Gender mainstreaming: an overview 2002 New York: United Nations

[B50] Department of Health Cancer Reform Strategy 2007 London: Department of Health

