# Supplementary material for: Men and women: beliefs about cancer and about screening
Source: BMC Public Health. 2009 Nov 24;9:431. doi: 10.1186/1471-2458-9-431 (PMC2789733; doi:10.1186/1471-2458-9-431)
Supplement: Additional file 1 — Questionnaire: Men and women: beliefs about cancer and about screening. Content of the questionnaire used for data capture. [file 1471-2458-9-431-S1.DOC]

# Men and women: beliefs about cancer and about screening

# Tracey H. Sach and David K. Whynes

# Content of the questionnaire used for data capture

*Note: The first page of the actual questionnaire contained a statement of the purpose of the research, an overview of the content of (and brief instructions for completing) the questionnaire, and full contact details.*

# What do you know about cancer ?

1. Approximately how many people are diagnosed with cancer each year in the United Kingdom ? (tick one box only):

| 125,000 | ⬜ _1_ | 200,000 | ⬜ _2_ | 275,000 | ⬜ _3_ |
| --- | --- | --- | --- | --- | --- |
| 350,000 | ⬜ _4_ | 425,000 | ⬜ _5_ | 500,000 | ⬜ _6_ |

1. Which is the most common cancer in the UK ? (tick one box only):

| Bowel (colorectal) | ⬜ _1_ | Breast | ⬜ _2_ |
| --- | --- | --- | --- |
| Cervical | ⬜ _3_ | Lung | ⬜ _4_ |
| Prostate | ⬜ _5_ | Skin (melanoma) | ⬜ _6_ |

1. Which of the following are risk factors for cancer ? (tick one box in each row):

|  |  | *Major risk* | *Minor risk* | *No risk* | *Don’t know* |
| --- | --- | --- | --- | --- | --- |
| (a) | Being overweight | ⬜ _1_ | ⬜ _2_ | ⬜ _3_ | ⬜ _4_ |
| (b) | Smoking tobacco | ⬜ _1_ | ⬜ _2_ | ⬜ _3_ | ⬜ _4_ |
| (c) | Too much alcohol | ⬜ _1_ | ⬜ _2_ | ⬜ _3_ | ⬜ _4_ |
| (d) | Lack of exercise | ⬜ _1_ | ⬜ _2_ | ⬜ _3_ | ⬜ _4_ |
| (e) | Certain sexually-transmitted infections | ⬜ _1_ | ⬜ _2_ | ⬜ _3_ | ⬜ _4_ |
| (f) | A family history of cancer (genetic) | ⬜ _1_ | ⬜ _2_ | ⬜ _3_ | ⬜ _4_ |
| (g) | Increasing age | ⬜ _1_ | ⬜ _2_ | ⬜ _3_ | ⬜ _4_ |
| (h) | Persistently high levels of stress and anxiety | ⬜ _1_ | ⬜ _2_ | ⬜ _3_ | ⬜ _4_ |

1. Are the following statements true or false ? (tick one box in each row):

|  |  | *True* | *False* | *Don’t know* |
| --- | --- | --- | --- | --- |
| (a) | “Sun-bathing can cause skin cancer”. | ⬜ _1_ | ⬜ _2_ | ⬜ _4_ |
| (b) | “Cancer is more common amongst women than it is amongst men” | ⬜ _1_ | ⬜ _2_ | ⬜ _4_ |
| (c) | “More people die of heart disease than die of cancer”. | ⬜ _1_ | ⬜ _2_ | ⬜ _4_ |

# Cancer screening

We are interested in your opinions as to the importance of three different types of cancer screening (listed in alphabetical order):

- **Bowel cancer**: Screening for bowel cancer can be accomplished either by analysis of faecal samples or by internal investigation (endoscopy). A national screening programme will be introduced in the near future. Bowel cancer affects both men and women, and around 34,000 new cases are diagnosed each year in the UK.
- **Breast cancer**: A national programme of screening for breast cancer has been operating for nearly 20 years. Screening entails a breast X-ray (mammography) every three years. Over 99% of breast cancers occur in women, and around 42,000 new cases are diagnosed each year in the UK.
- **Prostate cancer**: Whilst not presently organised as a national programme, screening for prostate cancer by means of a blood test (PSA) is available on request. Prostate cancer affects men only, as the prostate gland is part of the male reproductive system. Around 31,000 new cases are diagnosed in the UK each year.

1. For each of these three types of cancer screening:

|  | *Have you already had a screening test for this cancer ?*  *(tick if “yes”)* | *Would you take a screening test in the future, if one were to be offered ?*  *(tick if “yes”)* | *Tick if “not applicable”* |
| --- | --- | --- | --- |
| 1. Bowel | ⬜ _1_ | ⬜ _2_ | ⬜ _3_ |
| 1. Breast | ⬜ _1_ | ⬜ _2_ | ⬜ _3_ |
| 1. Prostate | ⬜ _1_ | ⬜ _2_ | ⬜ _3_ |

1. In general, do you think that the National Health Service ought to be providing cancer screening services ? (tick one box only).

Yes ⬜ _1_ No ⬜ _2_

# About you

1. Your gender: Male ⬜ _1_ Female ⬜ _2_
2. Your age (please write in): ………………………………………..years

1. Marital status – are you:

| Single | ⬜ _1_ | Married/living with a partner | ⬜ _2_ |
| --- | --- | --- | --- |
| Divorced or separated | ⬜ _3_ | Widowed | ⬜ _4_ |

1. Ethnic origin - please tick the box against the category which best describes you:

| African | ⬜ _1_ | Afro-Caribbean | ⬜ _2_ |
| --- | --- | --- | --- |
| Asian | ⬜ _3_ | Chinese | ⬜ _4_ |
| White | ⬜ _5_ | Other | ⬜ _6_ |

1. Your age on leaving full-time education:

(please write in) …………………………......years

1. Your annual household income (please tick one):

| Less than £10,000 | ⬜ _1_ | Between £10,000 and £20,000 | ⬜ _2_ |
| --- | --- | --- | --- |
| Between £20,000 and £30,000 | ⬜ _3_ | More than £30,000 | ⬜ _4_ |

1. Have you or a member of your close family ever suffered from the following (tick all those which apply):

| Stomach problems | ⬜ _1_ | Piles/ haemorrhoids | ⬜ _2_ |
| --- | --- | --- | --- |
| Heart disease | ⬜ _3_ | Cancer | ⬜ _4_ |
| Stroke | ⬜ _5_ | Depression | ⬜ _6_ |

1. Cigarette smoking – would you describe yourself as: (tick one)

| A smoker ? | ⬜ _1_ | An ex-smoker ? | ⬜ _2_ | Never smoked ? | ⬜ _3_ |
| --- | --- | --- | --- | --- | --- |

1. Your weight – would you describe yourself as: (tick one)

| Over-weight ? | ⬜ _1_ | About right ? | ⬜ _2_ | Under-weight ? | ⬜ _3_ |
| --- | --- | --- | --- | --- | --- |

1. How many times have you visited your GP in the past year? (please write in):
2. How many times have you visited your dentist in the past year? (please write in):

…………………………visits

…………………………visits

1. Compared with other men or women of your own age, do you think your chances of getting cancer are: (tick one only)

| *Much lower* | *Lower* | *The same* | *Higher* | *Much higher* |
| --- | --- | --- | --- | --- |
| ⬜ _1_ | ⬜ _2_ | ⬜ _3_ | ⬜ _4_ | ⬜ _5_ |

1. How worried are you about getting cancer ? (tick one only):

| Not at all worried | ⬜ _1_ | A bit worried | ⬜ _2_ |
| --- | --- | --- | --- |
| Quite worried | ⬜ _3_ | Very worried | ⬜ _4_ |

1. How would you describe your present state of health ? For each set of three options, please tick the one which best describes your health today.

| (a) | Mobility | I have no problems in walking about | ⬜ _1_ |
| --- | --- | --- | --- |
|  | (Tick one only) | I have some problems in walking about | ⬜ _2_ |
|  |  | I am confined to bed | ⬜ _3_ |
|  |  |  |  |
| (b) | Self-care | I have no problems with self-care | ⬜ _1_ |
|  | (Tick one only) | I have some problems washing or dressing myself | ⬜ _2_ |
|  |  | I am unable to wash or dress myself | ⬜ _3_ |

| (c) | Usual activities | I have no problems with performing my usual activities (e.g. work, study, housework, family or leisure) | ⬜ _1_ |
| --- | --- | --- | --- |
|  | (Tick one only) | I have some problems with performing my usual activities | ⬜ _2_ |
|  |  | I am unable to perform my usual activities | ⬜ _3_ |
|  |  |  |  |
| (d) | Pain and discomfort | I have no pain or discomfort | ⬜ _1_ |
|  | (Tick one only) | I have moderate pain or discomfort | ⬜ _2_ |
|  |  | I have extreme pain or discomfort | ⬜ _3_ |
|  |  |  |  |
| (e) | Anxiety and  depression | I am not anxious or depressed | ⬜ _1_ |
|  | (Tick one only) | I am moderately anxious or depressed | ⬜ _2_ |
|  |  | I am extremely anxious or depressed | ⬜ _3_ |

1. Compared with your level of health over the past year, is your health today:

| Better ? | ⬜ _1_ | Much the same ? | ⬜ _2_ | Worse ? | ⬜ _3_ |
| --- | --- | --- | --- | --- | --- |

1. Please feel free to add any further comments:
